# Unusual pathological fracture of the clavicle revealing primary hyperparathyroidism: a case report

**DOI:** 10.1186/s13256-017-1509-7

**Published:** 2017-12-09

**Authors:** Yassir Benameur, Hasnae Guerrouj, Imad Ghfir, Nouzha Ben Rais Aouad

**Affiliations:** Department of Nuclear Medicine, Ibn Sina Hospital, Mohammed V University, Rabat, Morocco

**Keywords:** Primary hyperparathyroidism, Bone scintigraphy, Parathyroid scintigraphy, Pathological fracture, Osteolytic bone metastases

## Abstract

**Background:**

Primary hyperparathyroidism revealed by a pathological fracture is very uncommon; in the majority of cases the discovery of lytic bone lesions on imaging examinations evokes in the clinician first a neoplastic etiology and a metabolic origin is often omitted. This case report adds to the existing literature as it describes an unusual presentation of primary hyperparathyroidism.

**Case presentation:**

We report a case of a 50-year-old Moroccan man, without any known tumor, who presented a fracture of his left clavicle with multiple osteolytic lesions on computed tomography suggesting bone metastases. However, bone scintigraphy oriented the diagnosis to a metabolic pathology by showing a metabolic bone “super scan” with increased tracer uptake in the left clavicle; parathyroid scintigraphy was able to localize pathological right parathyroid tissue.

**Conclusions:**

Whenever multiple osteolytic lesions are found in a patient without any known tumor, metabolic bone diseases including hyperparathyroidism should be highly considered.

## Background

Primary hyperparathyroidism (PHPT) is usually diagnosed as in incidental finding of raised serum calcium or due to symptoms secondary to the hypercalcemia such as polyuria, polydipsia, muscle weakness, gastrointestinal upsets, and renal stone formation [1].

The occurrence of bone lesions such as pathologic fractures in PHPT is now very rare since methods to measure serum calcium became available routinely in the mid-1970s [2].

We report a case of PHPT revealed by a pathological fracture of the left clavicle, associated with multiple osteolytic lesions which were initially confused with bone metastases. Bone scintigraphy oriented the diagnosis to a metabolic bone disease; elevated serum calcium and parathyroid hormone (PTH) confirmed the diagnosis of PHPT.

Our case report sheds light on a not commonly described presentation of PHPT revealed by bone complications, which were initially mistaken for bone metastases. We believe that the present case will contribute to available knowledge for the differential diagnosis of pathological fractures.

## Case presentation

A 50-year-old Moroccan man presented to our orthopedic department after tripping on a step and falling onto his left side. He had a 4-year history of type 2 diabetes and hypertension. His medication included furosemide, captopril, and metformin. He had a tobacco smoking history of 30 pack years, but he had quit smoking approximately 5 years ago.

A review of his social and environmental history did not indicate exposure to any other known toxins or carcinogens. He denied any family history of cancer. He is a police officer and he lives with his wife and two children.

A physical examination revealed a blood pressure at 135/80 mmHg, a normal pulse rate, and no fever; he had no symptoms except for the left clavicular pain.

**Fig. 1:**
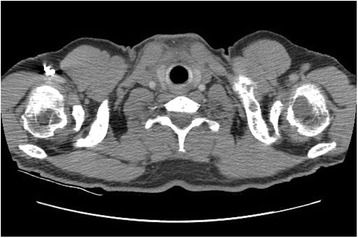
Thoracic computed tomography showing lytic lesions of the left clavicle and left humeral head and multiple lacunar foci

Radiographs showed a lytic lesion in his left clavicle and an asymptomatic lytic lesion in the left humeral head. A computed tomography (CT) scan was performed and objectified multiple osteolytic lesions on the whole skeleton (Fig. 1).

A neoplastic origin was first suspected; our patient was then referred to our nuclear medicine department with suspected bone metastases.

**Fig. 2:**
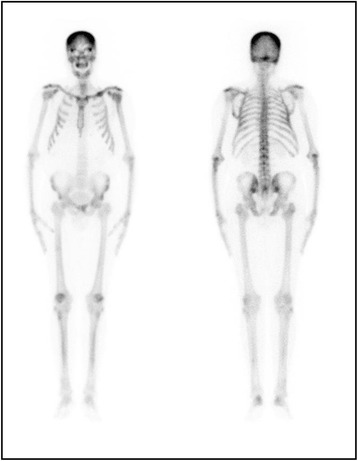
Bone scintigraphy with technetium-99m-methylene diphosphonate demonstrating diffusely increased uptake in both axial and appendicular skeleton, absent urinary activity, and prominent calvarian and periarticular uptake, with a focal accumulation of radiotracer on the left clavicle indicative of a metabolic bone disease

A whole-body bone scintigraphy with technetium-99m (^99m^Tc)-methylene diphosphonate (MDP) was particularly interesting insofar as it was able to orient the diagnosis to a metabolic bone disease. It demonstrated diffusely increased uptake in both axial and appendicular skeleton, absent urinary activity, and prominent calvarian and periarticular uptake, with a focal accumulation of radiotracer on the left clavicle (Fig. 2).

His complete blood count hemoglobin level was 15 g/dl, white cell count at 8900, and platelet count at 170,000; his transaminases and his serum electrolytes were normal. Hepatitis A serology, hepatitis B serology, hepatitis C serology, and human immunodeficiency virus (HIV) serology were also negative.

Laboratory studies also revealed an elevated serum calcium level of 3.8 mmol/l (corrected range, 2.15 to 2.60) and a circulating level of PTH of 1905 pg/ml (normal range, 14 to 72), his creatinine level was within normal limits. The diagnosis of PHPT was then confirmed.

**Fig. 3:**
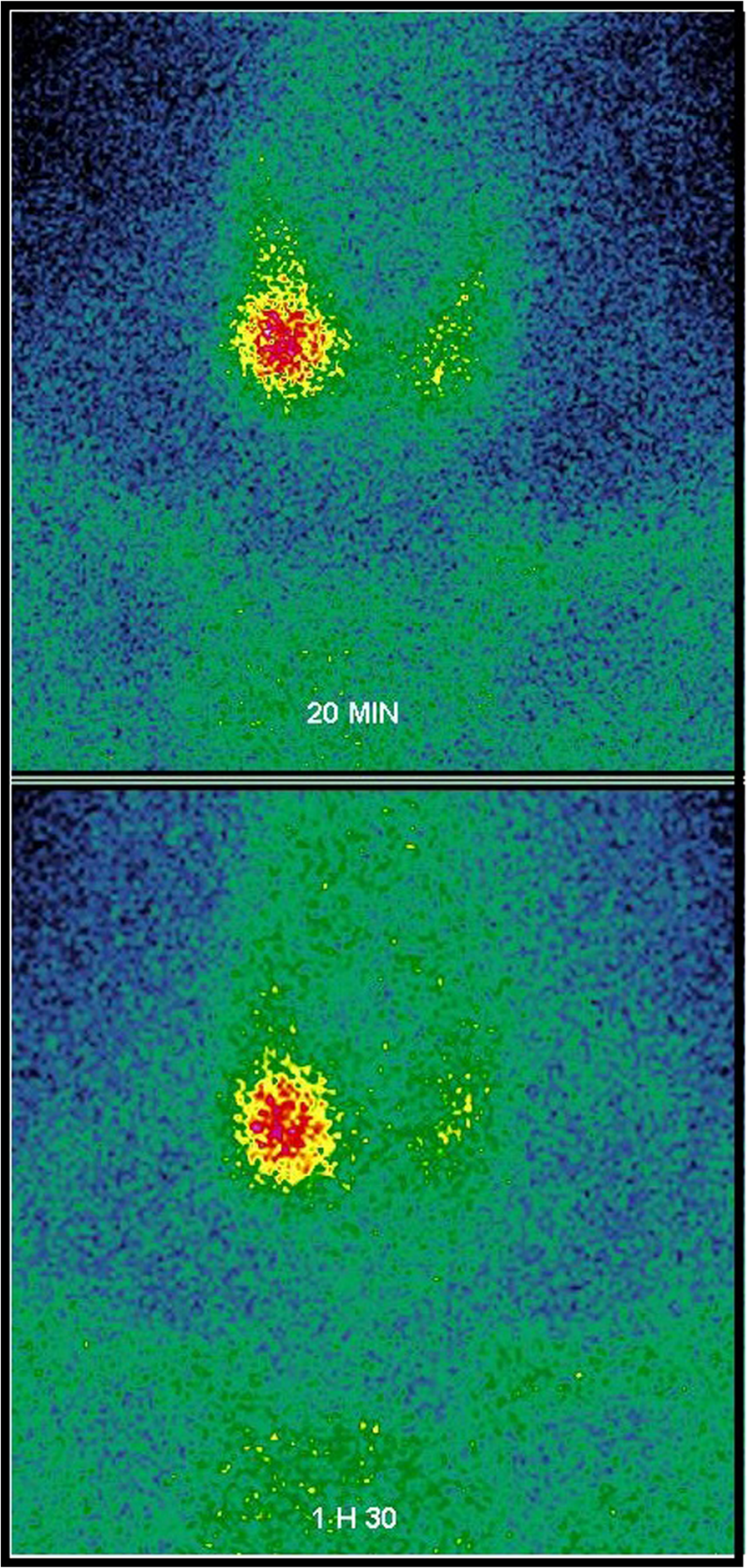
Parathyroid scintigraphy with technetium-99m methoxyisobutylisonitrile using washout method evoked pathological right parathyroid tissue

**Fig. 4:**
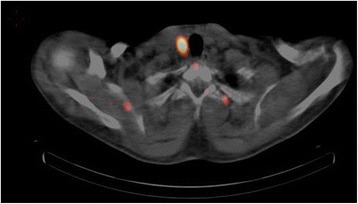
Axial fused cervical single-photon emission computed tomography/computed tomography image showing pathological right parathyroid tissue

Considering possible hyperparathyroidism caused by a parathyroid nodule, a cervical ultrasound was done and identified an adenoma in the right inferior parathyroid gland; this was confirmed by a parathyroid scintigraphy with ^99m^Tc-methoxyisobutylisonitrile (MIBI) using washout method, which showed a focal increased uptake in inferior right parathyroid gland (Figs. 3 and 4).

A parathyroidectomy was performed and a large parathyroid tumor was removed and confirmed to be a benign parathyroid adenoma on histological examination.

After surgery, our patient’s PTH level returned to normal but he developed hypocalcemia, requiring doses of orally administered calcium. He was alive and showed no symptoms during a 6-month follow-up period.

## Discussion

Before undergoing the bone scintigraphy, our patient was suspected to have bone metastases. Fortunately, the bone scan demonstrated an aspect of metabolic disease; his serum calcium and PTH levels led to the correction of the diagnosis to PHPT.

This case highlights that pathological fracture is not always synonymous with cancer, several differential diagnoses must be taken into consideration, among them PHPT.

PHPT is a relatively common endocrine disorder, with prevalence estimates of one to seven cases per 1000 adults. It is believed to be the most common cause of hypercalcemia, predominantly affecting elderly populations and women two to three times as often as men. The incidence of PHPT has been difficult to assess, available estimates vary widely from 0.4 to 21.6 cases per 100,000 person-years [3].

In most patients with PHPT (80 to 85%), the disease develops due to a parathyroid adenoma, in 15 to 20% due to parathyroid gland hyperplasia, and in < 0.5% due to parathyroid carcinoma [4].

Many cases of PHPT are diagnosed in the early stages of the disease process, before the development of classical clinical findings of prolonged disease such as nephrolithiasis, brown tumors or osteitis fibrosa cystica, bone cysts, or pathologic fracture. Early diagnosis has resulted in a significant decline in what were once the common morbidities of the disease [5].

The incidence of pathological fractures in PHPT is relatively rare accounting for around 10% in two large series [6]. However, PHPT revealed by pathological fracture, as in our case, is very uncommon.

Morgan *et al*. reported a case of subtrochanteric fracture of the femur through an area of lytic bone with hypercalcemia which was felt to be a metastatic pathological fracture [7], the same presentation was found in the case reported by Khaoula *et al*. [2]. Rachha also reported three cases which involved the long bones [1].

A literature review of other reported cases using the PubMed database revealed that our case is the first presentation of PHPT revealed by clavicle fracture.

A cancerous origin is suspected before a pathological fracture with multiple lytic bone lesions; this was the situation in our patient, who underwent skeletal scintigraphy to look for bone metastases. However, fortunately, the bone scan showed a feature of metabolic bone disease. With metabolic bone disease there is a generalized increased uptake throughout the skeleton and markedly reduced soft tissue background activity. The kidneys appear faint or there may be absence of the normal renal activity, giving the “super scan” because of the predominance of the bone images [8]. PHPT is one of the main etiologies of this feature on bone scintigraphy. Other skeletal anomalies secondary to hyperparathyroidism are also possible and are detailed in Table 1 [9–12].

**Table 1:** All skeletal and extraskeletal anomalies secondary to hyperparathyroidism

Skeletal anomalies	Early stage: • Symmetrical abnormal bone activity is present, particularly in the calvarium, mandible, sternum, and epiphyses of long bones with or without accompanying changes in the vertebrae and pelvis [9].
	Severe cases: appearances are those of a *super scan*: • Increased tracer uptake in axial skeleton. • Increased tracer uptake in long bones. • Increased tracer uptake in periarticular areas. • Faint or absent kidney images [10].
Extraskeletal anomalies	Extraskeletal metastatic calcification in primary hyperparathyroidism: • Increased visceral uptake of technetium-99m-labeled phosphate throughout the lung, liver, stomach [11].
	Extraskeletal anomalies secondary to secondary hyperparathyroidism: • Increased diffuse activity in the lungs and the small kidneys. • Increased muscle activity (diffuse and bilaterally in the vast and adductor of both thighs, and in the abdominal muscles) [12].

Once the diagnosis of PHPT is confirmed by increased serum PTH level, parathyroid localization studies such as neck ultrasound scan and ^99m^Tc-MIBI scintigraphy are used to confirm the location of the diseased gland rather than for establishing the diagnosis of PHPT preoperatively.

Initially, ^99m^Tc-MIBI is concentrated in the thyroid gland and abnormal parathyroid tissue and then differentially clears from these organs in a time-dependent manner; washout of the thyroid gland is more rapid than from the abnormal parathyroid tissue that allows dual-phase ^99m^Tc-MIBI scintigraphy to detect the hyperfunctioning parathyroid tissue [5].

Parathyroidectomy is the treatment of choice in PHPT. In the last 10 to 15 years, surgery for PHPT has moved from the wide bilateral neck exploration to minimally invasive approaches, such as the minimally invasive radio-guided parathyroidectomy [13].

## Conclusions

This case illustrates a rare presentation of a parathyroid adenoma revealed by clavicle fracture, and highlights the importance of explorations using nuclear medicine. When a patient presents with unexplained osteolytic lesions, although metastatic malignancies should be ruled out first, other differential diagnoses such as metabolic bone disease including hyperparathyroidism should be kept in mind as well, in order to reach an accurate diagnosis.

## Abbreviations

^99m^Tc: Technetium-99m
CT: Computed tomography
HIV: Human immunodeficiency virus
MDP: Methylene diphosphonate
MIBI: Methoxyisobutylisonitrile
PHPT: Primary hyperparathyroidism
PTH: Parathyroid hormone
